# Multi-objective database queries in combined knapsack and set covering problem domains

**DOI:** 10.1186/s40537-021-00433-x

**Published:** 2021-03-10

**Authors:** Sean A. Mochocki, Gary B. Lamont, Robert C. Leishman, Kyle J. Kauffman

**Affiliations:** 1 Department of Electrical and Computer Engineering, Air Force Institute of Technology, 2950 Hobson Way, Wright-Patterson Air Force Base, 45433 USA; 2 Integrated Solutions for Systems, 4200 Colonel Glenn Highway, Beaver Creek, 45431 USA

**Keywords:** Genetic Algorithm, Hill Climber Algorithm, Knapsack Problem, Set Covering Problem, Position Navigation and Timing, The Knapsack Set Covering Problem, Problem Domain, Algorithm Domain, Multi-Objective

## Abstract

Database queries are one of the most important functions of a relational database. Users are interested in viewing a variety of data representations, and this may vary based on database purpose and the nature of the stored data. The Air Force Institute of Technology has approximately 100 data logs which will be converted to the standardized Scorpion Data Model format. A relational database is designed to house this data and its associated sensor and non-sensor metadata. Deterministic polynomial-time queries were used to test the performance of this schema against two other schemas, with databases of 100 and 1000 logs of repeated data and randomized metadata. Of these approaches, the one that had the best performance was chosen as AFIT’s database solution, and now more complex and useful queries need to be developed to enable filter research. To this end, consider the combined Multi-Objective Knapsack/Set Covering Database Query. Algorithms which address The Set Covering Problem or Knapsack Problem could be used individually to achieve useful results, but together they could offer additional power to a potential user. This paper explores the NP-Hard problem domain of the Multi-Objective KP/SCP, proposes Genetic and Hill Climber algorithms, implements these algorithms using Java, populates their data structures using SQL queries from two test databases, and finally compares how these algorithms perform.

## Introduction

Big Data is a field that has been receiving a lot of attention in recent years. While a definitive definition is elusive, a definition which provides context is that big data is “the datasets that could not be perceived, acquired, managed, and processed by traditional IT and software/hardware tools within a tolerable time” [1]. This definition implies that Big Data involves such quantities of data that non-traditional methods are necessary for managing this data. A recent example of the application of Big Data with respect to world events was when Taiwan combined its health insurance and immigration and customs databases to perform analysis to help characterize and control the spread of COVID-19 [2].

As a specific instance of a Big Data database, consider a relational database that is designed to organize Position, Navigation and Timing (PNT) metadata specifically related to test flights conducted by the Air Force Institute of Technology (AFIT). AFIT has approximately 100 data logs (also called missions) which will be stored in this relational database, and this number is expected to grow annually. This database requires a number of queries from parties interested in using this data for research.

Two relevant well-known Nondeterministic Polynomial (NP)-Complete problems are the 0/1 Knapsack Problem (KP) and the 0/1 Set Covering Problem (SCP) [3–6]. The terms 0 and 1 indicate that, given a Problem Domain (PD) of objects, each object is either included in a specific solution (1), or excluded (0). An answer in this pd is one that meets all of the problem constraints, and a global optimum is one that has a better objective function then all other solutions in the search space [7].

Consider the following brief description of the combined Multi-Objective (MO) KP/SCP when converted to a database query: “Return the set of data logs where at least one data log meets n different criteria, and for which at least one each of x category are utilized with the summation of z number totaling no more than c number, and maximizing k.” A specific implementation of this query may be: Return the set of data logs where a given set of sensor types and terrain types are represented across the set of returned data logs (and every data log contributes at least one sensor or terrain type not contributed by a separate log), and where the number of sensor readings recorded over 1000 meters in altitude across all data logs is maximized, and the total summed time of all data logs does not exceed 2 h.” Section "Methods/Experiments" of this paper describes the combined MO KP/SCP PD. This problem is NP-Hard (A proof is available in the appendix of Mochocki [8]).

Various stochastic algorithms have recently been used to solve the SCP. One example is the Black Cat Swarm Optimization (BCSO) algorithm, proposed by Crawford et al. [9], which was based on the earlier Cat Swarm Optimization (CSO) algorithm defined in Chu et al. [10] and adapted to solve discrete optimization problems by Sharafi et al. [11]. Another frames the transit route network design problem TrNDP as a MO optimization problem, and uses the Route Constructive Genetic Algorithm (RCGA) to translate it into a SCP [12], which is then solved using randomized priority search as defined in Lan et al. [13]. Additional algorithms used to solve the SCP are the black hole algorithm [14, 15], the Harmony Search Algorithm [16] and the hyperedge configuration checking strategy [17] .

Numerous Genetic Algorithm (GA)s exist in recent literature which solve the KP [18–21]. Ezugwu et al. [22] performed a comparative study of various approaches to the 0/1 Knapsack problem, including GAs and simulated annealing. An additional swarm based algorithm used to solve the KP is the moth swarm algorithm [23], and the fruit fly algorithm [24].

There are many examples in the literature of GAs being used to solve a Multiobjective Optimization Problem (MOP) [7, 25–29]. Due to the complexity of the PD and the recorded success of GAs used with MOPs, this paper proposes a GA stochastic population based search algorithm and a Hill Climber (HC) stochastic local search algorithm to return answers to the combined KP/SCP PDs. Both are based on a GA developed by Beasley and Chu [30], and utilize modified code based on work by Cobonkerr [31]. These algorithms are relatively simple and are not expected to push the field of GAs forward significantly, but rather to show that they can be used with a novel database in a unique PD to generate information useful for researchers. A literature review did not discover other studies with comparable results to those laid out here, nor did it uncover the combination of the KP and SCP.

The various modern SCP and KP algorithms referenced in the prior paragraphs could also be adapted to solve the combined KP/SCP. Comparing the results in this paper to the performance of these other algorithms is difficult, because the results in this paper are based on querying a non-standard database with randomized data, populating data structures and further randomizing this data, and then running the GA and HC algorithms developed by Beasley and Chu [30]. Even so, the results of this experiment are compared to the BCSO in Section "Discussion" and demonstrate that these algorithms are competitive when trading off answer quality and speed. If the use of GAs with database queries proves to be useful, additional effort can be made to apply these more modern stochastic algorithms to the field of database MOPs.

These algorithms are tested against two relational databases, one with 100 navigation data logs, and one with 1000 data logs. These databases are populated with six files of repeated sensor data in the Scorpion Data Model (SDM) format, which is discussed in Section "Methods/Experimental", and randomly entered metadata. They are implemented in Java, and use Structured Query Language (SQL) queries to populate Java data structures, which then run the algorithms and return answers to the NP-Hard combined MO KP/SCP. In application, this Java program functions as part of the User Interface, and would interact with the underlying database on behalf of the researcher. The benefit is that the queries are simple, but allow for more complex results to be returned. The queries in the next section could modified at will. As long as the input to the MO KP/SCP implementations fits the format defined in this paper, it allows for any sub-queries to populate the relevant problem data structures.

Section "Background and related queries" of this paper discusses the background of the relational databases used to test the stochastic algorithms. Section "Problem Domain" discusses the PD of the SCP and the KP, and the combined KP/SCP. Section "Stochastic Algorithms for the Combined MO KP/SCP" develops the pseudo code for the GA and the HC. Section "Results" presents the test experiment and results, and Section "Discussion" compares them to a modern SCP algorithm. Section "Conclusion" is the paper conclusion, and Section 6 is the authors’ declarations.

## Methods/experimental

This section is divided into three parts. Section "Background and related queries" describes the background of the database, and which queries are used in order to construct the combined SCP/KP. Section "Problem Domain" goes into detail on the SCP, KP, and the combined SCP/KP PDs. Section "Problem Domain" describes the two algorithms developed to solve the combined KP/SCP, and Section "GA and HC algorithm expected performance" describes the anticipated algorithm performance based on an analysis of the two algorithms and the PD

### Background and related queries

This section covers some specific details of the database design in order to motivate how the SCP and KP can combine to help facilitate database queries. This database is implemented in PostgreSQL, which is an open source, object-relational database system which dates back to 1986 at the University of California at Berkely. This Relational Database Management System (RDBMS) supports arrays and documents (such as JavaScript Object Notation (JSON)), is also extensible, in that additional data types, functions, and operators can be added [32].

**Fig. 1:**
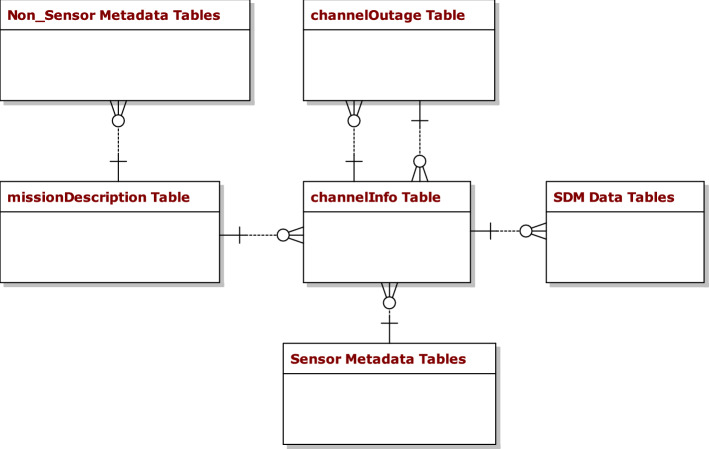
Relational PNT Database Overview: Every row in missionDescription Table is an independent data log in the SDM format which is uploaded in the database. The SDM tables contain all of the navigation data associated with the data logs

Three potential PostgreSQL relational database designs are compared to store PNT data [8]. These designs offer identical ways to store sensor metadata and non-sensor metadata, and differing ways to store sensor data which is recorded in the SDM format. A database overview from [8] and [33] is shown in Fig. 1. Figure 1 is a simplification of the actual design of the database. Wherever table is plural (i.e. tables), there are numerous tables, each which have their own relationships to each other. For additional detail on the underlying database design and on how the final design was selected, please reference [33]. For this paper, the queries were kept relatively simple in order to demonstrate the utility of the algorithms, but more complicated queries could be used as long as they returned data matching the format described in the algorithms:

The missionDescription table in the overview above depicts the various missions stored in the database. In other words, a row in the missionDescription table, along with all of the other data related to that row in the children tables, together comprise all of the available information for that mission. The Sensor Metadata table is coupled with the channelInfo table so that specific sensors can be affiliated with channels for a given mission, and the SDM Data tables are comprised of the data collected by those sensors.

For the chosen approach, a new table is created for each SDM data type identified in Table 1 and for each mission. As an example, an individual row in the altitude table will have: artifact data for the relational database, and data which stores the measured SDM altitude data. This measured data includes: altitude (Double Precision), variance (Double Precision), timeStamp_arrival_sec, (BIGINT), timeStamp_arrival_nsec (INT), timeStamp_valid_sec (BIGINT), and timeStamp_valid_nsec (INT). Consult [34, 35] for additional detail on the SDM. [8] goes into additional detail on the tests which discriminated between these three solutions, provides rational for the solution chosen to meet AFIT’s data storage needs, and provides the specific SQL script which generates these tables.

**Table 1:** SDM Data Types: Describes the standardized types of sensor data recorded in each data log

SDM Data Types	Description
IMU	Inertial Measurement Unit (IMU) delta velocity and delta rotation measurements from the device’s three axis accelerometers and three axis gyroscopes.
Velocity (3D)	3-dimensional velocity
Velocity (1D)	1-dimensional velocity
Speed	Speed as the magnitude of the velocity vector
Altitude	Height above the WGS-84 ellipsoid
GeodeticPosition (3D)	3D WGS-84 GeodeticPosition
ThreeAxisMagnetometer	Measures magnetic field as a 3-dimensional vector
PositionVelocityAttitude	Includes position, velocity, rotation, and uncertainty
OpticalCameraImage	Image from an optical camera
GNSS	Raw measurements from a Global Navigation Satellite System (GNSS) receiver
GPSEphemeris	Ephemeris describing GPS satellite locations.
SensorRegistrationAck	Message to register sensor
non_SDM_Message	Lightweight Communications and Marshalling (LCM) message is not an SDM data type

A new table is created for each data type and for each data log. The IMU table, for instance, would have 430 Million rows for the 1000 mission database if they were not split between tables

For each approach, two databases were created, one with 100 missions and one with 1000 missions, in order to help show how these approaches scale as they become larger. The missions are composed of six log files in the LCM format, which are uploaded repeatedly into the databases, along with randomized sensor and non-sensor metadata which are associated with each data log.

In order to populate the data structures discussed later in this article, a series of SQL queries are used in conjunction with the PNT database. The following queries are written for the chosen database design and are discussed here, along with their use in populating data for this algorithm: 
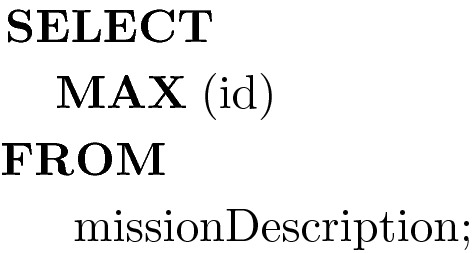


Purpose: Every mission is a column which may be part of a returned solution. This query identifies the number of missions in the database and updates the total number of columns in the problem based on this number 
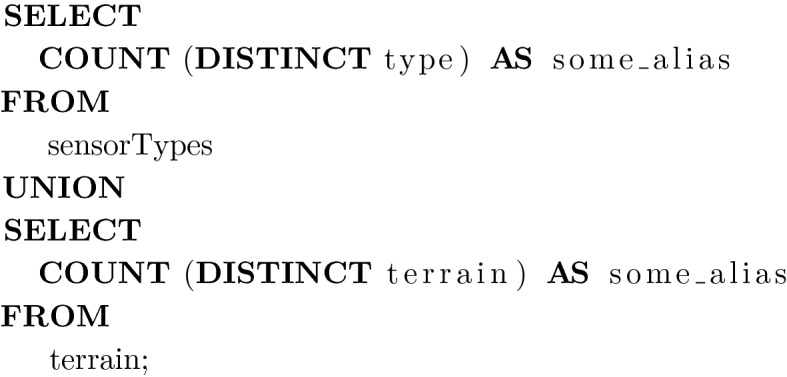


Purpose: This query determines the number of sensor types and terrain types to be covered. This returns the number of rows 
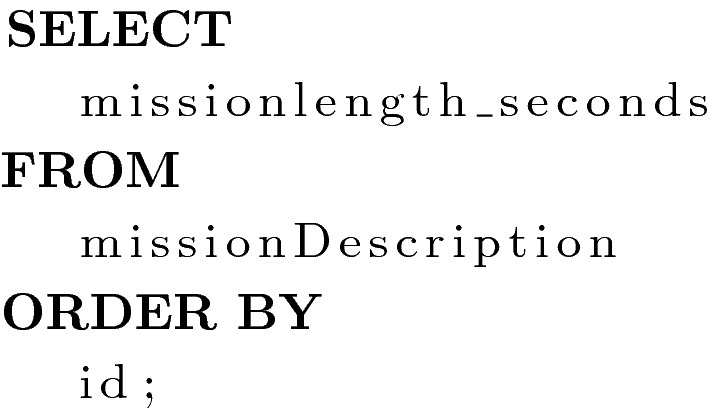


Purpose: This query returns the weight of each column for the combined KP/SCP 
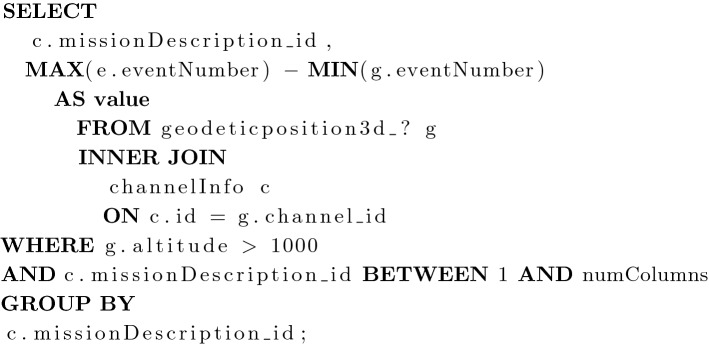


Purpose: This query returns the value of each column for the combined KP/SCP. The GeodeticPosition (3D) data type is chosen to determine the minimum and maximum event numbers because it is a common data type which exists across most missions 
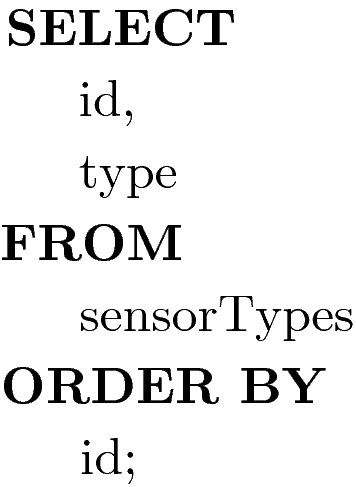


Purpose: This query returns a list of sensor types and their associated mission identifiers. These are the first eight rows to be covered by the KP/SCP 
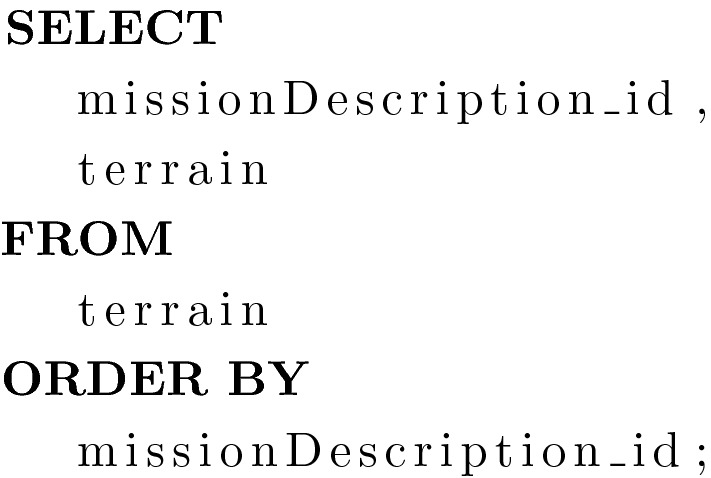


Purpose: This query returns a list of terrains and their associated mission identifiers. These are the last 5 rows to be covered by the KP/SCP.

These queries take time and have an overhead observable in the testing data. They take approximately 2 s to complete for the 100 mission database, and 17 s to complete for the 1000 mission database. This will help contextualize the performance of the GA and HC algorithms. The next two sections of this paper discuss the combined KP/SCP PDs, and the GA and HC algorithm.

### Problem domain

In order to define how the problem space of the SCP and KP combine, each one is defined individually. Following this, their combination is addressed.

#### Set covering problem

The general SCP entails having a group of sets which are to be covered (defining the universe), and a group of families, each which is composed of a subset of sets, so that a complete covering of the sets is composed of a group of families, the combined sets of which include all of the sets to be covered. The SCP is defined as [36]:

$$\begin{aligned}& \text {Set Covering Problem: Given a set } R = \{r_1,\ldots, r_m\} \text { and a family } F = \{S_1,\ldots,S_N\} \\ & \text { of sets } S_j \subset R \text { any subfamily } F^{\prime} = \{S_{j1},S_{j2},\ldots S_{jk}\} \text { of F such that } \cup _{i=1}^kS_{ji} = R \\ & \text { is called a subset covering of R, and the } S_{ji} \text { are called the covering sets.} \end{aligned}$$

The SCP can be thought of in terms of [3]:

$$\begin{aligned} J_i = \{j\in J:a_{ij}=1\} \\ I_j = \{i\in I:a_{ij}=1\} \end{aligned}$$

which describes the columns which cover the rows and the rows which are covered by the columns. This concept is applied in code by defining two dimensional array lists in Java.

Typically the SCP is thought of as having costs such that the goal is to Minimize

$$\begin{aligned} \sum _{j\in J} c_jx_j \end{aligned}$$

which means that the families chosen to be part of the solution have associated costs which are to be minimized. This minimization is part of the optimization process.

*Solution space* The SCP is ${O(2^{n})}$ [37]. This is due to a full solution having to consider all possible combinations of the available sets.

*Problem class* NP-Complete [5].

The SCP can be framed as a query for the PNT database described in Sections "Introduction" and "Methods/Experimental": Return a subset of data logs that minimize the total summed time of the missions such that all terrain types and sensor types are represented.

#### The knapsack problem

In the general form of the KP problem, objects have an associated weight and value which are not (necessarily) related, and bins have a certain weight capacity. An optimal answer returns the set of items that fit in this bin that have the maximum value.

$$\begin{aligned}& \text { Knapsack Problem: Given a set of } n \text { items. Each item } i = 1\ldots n \text { has two parameters, } \\& \text { a weight } w_i \text { and a value } v_i \text {. Given a knapsack capacity } X \text {, find a subset } X \text {of items} \\& \text { of maximum value that does not exceed the weight restriction. The goal is to} \\& \text {maximize } \sum _{i \in S} v_i \text { such that } \sum _{i \in S} w_i \le X { [5].} \ \end{aligned}$$

*Solution space* The KP is ${O(2^{n})}$. This is due to a full solution having to consider all possible combinations of the available sets [5].

*Problem class* NP-Complete [5].

The KP can be framed as a query for the PNT database described in Sections Introduction" and "Methods/Experimental": Return a subset of data logs where the total summed time of missions does not exceed X s, and where the total number of sensor measurements taken above Y altitude are maximized.

#### Combined problem

Find a solution such that both problem domains are satisfied and optimized. SQL queries are used against the PNT database to populate data structures affiliated with the combined KP/SCP. These data structures are used with conjunction with a Population (GA) and Local Search (HC) algorithm to return answers to the queries in the combined KP/SCP problem domains.

*English Description* Given a knapsack capacity, a set of rows to be covered, and a set of families which each have some subset of these rows, and also have an associated value and weight. Return a subset of these families which covers all of these rows (combinatorial problem, one type of optimization), and for which the combined weight does not exceed the knapsack capacity (constraint), and which has the maximum possible combined value for the sets (optimization).

$$\begin{aligned}& \text {The KP/SCP: Given a capacity } X \text {, a set } R = \{r_1,\ldots r_m\} \text {, and a family } F = \{S_1,\ldots S_N\} \\ & \text {of sets } S_j \subset R \text { and associated weights } F_w = \{w_1,\ldots, w_N\} \text { and values } F_v = \{v_1,\ldots v_N\} \text {,} \\ & \text {return a subfamily } F^{\prime} = \{S_{j1},S_{j2},\ldots S_{jk}\} \text { of } F \text { such that } \cup _{i=1}^k S_{ji} = R \text {, which } \\ & \text {maximizes } \sum _{i \in F} v_i \text { such that } \sum _{i \in F} w_i \le X \text { and for which } \not \exists [(S_n)|S_n \subset (F^{\prime} - S_n)] \end{aligned}$$

The goal in this problem domain is to optimize the combined value

1 $$\begin{aligned} \sum _{i \in F} v_i \end{aligned}$$

and to provide a set covering such that

$$\cup _{i=1} ^k S_{ji} = R$$

The weight

$$F_W = \{w_1,\ldots w_N\}$$

can be thought of in terms of the cost from the original SCP problem, but this is not a perfect corollary. For instance, it does not matter if

$$\begin{aligned} \sum _{i \in F} w_i = X \end{aligned}$$

or if the combined weight is arbitrarily lower, so just trying to minimize weight is not necessarily an optimization goal. The goal is to provide a set covering so that value is maximized, while not exceeding the aforementioned constraints. These are competing optimizations, as there may be solutions to the Knapsack Problem which have higher values yet which do not provide a set cover, and there may be smaller set coverings with lower weights which provide less value.

*Solution space * Both of these problems could be solved independently if the relevant parameters from the other problem were ignored. Even so, each is a permutation problem, resulting in them having equivalently sized solution spaces. Therefore this problem has a solution space of ${O(2^{n})}$, which is the same as if they were solved independently.

*Problem class* The KP/SCP PD can be thought of as a decision problem. For a given answer to a SCP, is it a valid minimal set cover that meets the three combined conditions:

2 $$\begin{aligned}&\cup _{i=1} ^k S_{ji} = R \end{aligned}$$

3 $$\left[ {\left( {S_{n} } \right)\left| {S_{n} \subset \left( {F^{\prime} - S_{n} } \right)} \right.} \right]$$

4 $$\begin{aligned}&\sum _{i \in F} w_i \le X \end{aligned}$$

The SCP decision problem reduces to the combined KP/SCP Problem. A proof is available in [8]. This shows that the KP/SCP decision problem is NP-Complete. The KP/SCP problem is likely NP-Hard, as it would be difficult to provide a polynomial time certifier that a given answer is indeed optimal. The next section describes the top down design of algorithms to solve the combined MO KP/SCP. The SQL queries in Section "Methods/Experimental" are used to populate data structures, which are used with these algorithms to return answers to this PD.

### Stochastic algorithms for the combined MO KP/SCP

The code for the GA and HC algorithms sections are based on the work by Cobonkerr [31], which implements the algorithm designed by Chu and Beasley [38]. Some sections of the code required major rewrites to implement the modified algorithms. The pseudo code is available in the appendix of Mochocki [8].

#### Genetic algorithm

Consider the general description of a GA [39]:

1. Initialize a population of chromosomes; (Set of Candidates)
2. Evaluate each chromosome in the population; (Feasibility)
3. Create new chromosomes by mating current chromosomes—apply mutation and recombination as the chromosome mate; (Next State Generator)
4. Evaluate the new chromosomes (Feasibility) and insert them into the population; (Objective Function)
5. If time is up, stop and return the best chromosome; (Solution), if not, goto 3.

Encoding: Utilize a binary encoding (0/1) for each column to indicate whether or not that specific column is considered as part of a given solution. Each column has an associate weight and value. A specific solution is represented as a genome (equivalent to a chromosome).

Initial Population (Set of Candidates): This is generated at the beginning of the program based on the specific column/row/weight/value parameters. Each member of the population is checked for feasibility, as detailed below, and to ensure lack of redundancy between answers.

Training Solutions (Next State Generator):

- Use a k-ary tournament selection to select two parents, each is the most fit of its respective tournament (participants are randomly selected) [7]. This approach gives preference to the more fit parent, but does not guarantee that a bit from that parent is selected.
- Perform a crossover between the two parents to produce a child. Consider each bit in parents. If bits match, pass bit to child. If bits do not match: Generate fitness number:${f_{prob} = }\, \frac{f_{p2}}{f_{p1} + f_{p2}}$. Generate a random number *r* with the range ${0\ldots(f_{p1} + f_{p2})}$.
- if ${r > f_{prob}}$ take the bit from ${p_{2}}$ else ${p_{1}}$.
- Perform a mutation on the child to produce a new solution. Select a random bit from child and flip
- Check the child for feasibility, perform a modification on the child to make feasible
- Add the child back to the population, replacing a less fit member. Calculate average fitness of population ${p_{a} = (\sum _{i=1}^{n}v_{i} \frac{1}{n})}$. Randomly choose a member of population. If ${v_{m}\le \, p_{a}}$ replace, else choose a new member.

Feasibility: The feasibility of the population is checked once generated, and every new possible solution is checked before being returned to the population.

- Per the SCP, the set of rows which are part of the solution need to be checked to confirm that every row is covered.
- The answer needs to be a minimal subset so that there is no column for which every covered row is a subset of the rows covered by all of the other combined columns.
- The combined weight of all columns needs to not exceed the weight restriction.

Mathematically, feasibility is defined as:

1. $$\cup _{i=1} ^k S_{ji} = R$$
2. $$\not \exists [(S)|S \subset (F^{\prime} - S)]$$
3. $$\sum _{i \in F} w_i \le X$$

In the case a given solution is not feasible, it is fixed or discarded deterministically. Note, it is assumed that Feasibility Condition 2 can only occur if Feasibility Condition 1 is valid. In other words, a solution cannot be a minimal SCP if it is not an SCP.

Make Feasible: Consider cases in Table 2

**Table 2:** Genetic algorithm possible feasibility conditions

Case	1	2	3
a	T	T	T
b	T	T	F
c	T	F	T
d	T	F	F
e	F	T	T
f	F	T	F
g	F	F	T
h	F	F	F

Case Descriptions:

- All three conditions are true. This means that the solution is feasible. Return genome.
- Min SCP conditions are satisfied but KP condition is not. Discard this genome.
- Answer is a SCP and satisfies KP, but is not minimal. Identify redundant family of lowest value (if multiple) and delete. Return modified genome.
- Answer is a SCP but there are redundant columns and KP weight condition is violated. Identify redundant columns and remove so that weight falls within restriction. If weight still exceeds limit even once all possible columns are removed, discard this genome.
- The solution is not a set cover but the columns proposed so far do not violate weight limits. See if any columns can be added which meet condition 1 without violating the weight restriction. Then check for redundancy with added rows.
- The solution is not a set cover and it busts the weight limits. Discard this genome.
- Condition 2 assumes that condition 1 is valid, so this combination is meaningless.
- Condition 2 assumes that condition 1 is valid, so this combination is meaningless.

Fitness (Objective Function): Assuming that all solutions are feasible, the best answer, also called the most fit, is the one with the highest value.

Convergence:

1. Hard time restriction: This value can be set as a problem parameter and sets a limit to for how long the algorithm will continue to hunt for better solutions even while these solutions are actively being found. This time restriction is only checked in between attempts to evolve new solutions, it will not interrupt an evolution attempt.
2. Population fitness has not changed in at least 60 iterations. This implies that a more fit solution is relatively difficult to find.

#### Hill Climber Algorithm

The Hill Climber Algorithm uses the same problem encoding, queries, and generation of the initial solution as the GA. The main difference is that the HC only generates a single solution, and then explores the neighborhood of that solution looking for better answers, terminating when it is completely explored.

Encoding: Use a binary encoding (0/1) for each column/item to indicate whether or not that specific column is considered as part of a given solution. Each column/item also has an associated weight and value.

Neighborhood: A given subfamily $F^{\prime} = \{S_{j1},S_{J2},\ldots,S_{jk}\}$ of F such that $\cup _{i=1}^kS_{ji}=R$ and feasibility constraints are met, subject to all possible Swap combinations $O(n^2)$.

Initial Population (Set of Candidates): A single solution $S_0$ is generated as the initial solution. The generation of this solution matches the process described in section “Genetic algorithm”.

Next State Generator: Utilize swap operation. $\forall F(S_1,\ldots,S_N)$ if $S_j=$“1” and $S_k$ = “0” Swap $(S_j,S_k)$. Then check for feasibility

Feasibility: The solution encoding is identical to section “Genetic algorithm”. Therefore, the constraints and challenges associated with the feasibility of a particular swap operation are the same. The feasibility function returns either the $\emptyset$ or $S_{jn}$.

Selection: If $F_{t+1} > F_t$, Make $S_{t+1}$ the new current state, else, discard $S_{t+1}$ and generate a new next solution per Next State Generator

Solution: Terminate either when: $t=T$, i.e. a defined amount of time has elapsed or entire neighborhood has been explored and no better solution returned.

Fitness (i.e. Objective Function): Assuming that all solutions are feasible, the best answer (i.e. most fit) is the one with the highest value.

### GA and HC algorithm expected performance

Section "Stochastic Algorithms for the Combined MO KP/SCP" laid out the background for the GA and the HC pseudo code. The key difference between them is that the GA uses a population based approach, k-ary tournament selection, a fitness-based crossover, mutation, and returns the genome to the population based on a fitness comparison with the genome to be replaced. The GA does not run the risk of getting stuck in a single neighborhood, even though the fittest answers may trend towards a local maximum, and not reach the absolute maximum which may be somewhere else in the solution space, especially if the crossover and mutation operators alone are not able to get any of the genomes in the population there.

The Hill Climber explores a specific neighborhood, which is defined as the swap of all bits for a randomly generated started genome. The make feasible function of the GA is used only for the generation of the initial solution, any non-feasible solutions for the HC are discarded, even if they might potentially lead to more fit answers. Functionally, the HC uses much of the structure of the GA.

Based on their design, we expect that the GA will in general produce more fit answers to the combined MO SCP/KP in comparison to the HC. The GA is not restricted to a particular neighborhood, and multiple neighborhoods may be represented in the population. The HC is expected to find its solutions faster than the GA. Once the initial solution is discovered, the HC deterministically searches its neighborhood in polynomial time, whereas the GA stochastically generates the entirety of its initial population before beginning to the genome mutation process.

In Section "Results", the GA is tested with populations of 10, 25, and 50 genomes for the 100 and 1000 mission databases. The PD search space represented by the two databases used to test these algorithms is critical when comparing how the GA performs between population sizes. As is discussed in the next section, these databases are composed of repeated files and randomized metadata, with values and weights stochastically modified after being queried to add nuance to the search space. Even so, certain combinations of files with high value and low weight are expected to be present in most answers, especially as the weight limits increase, so it is expected that GAs with population sizes of 50 will produce similarly fit solutions as the GAs with a population sizes of 10. In general, for populations that use a binary encoding smaller population sizes are sufficient to find relatively fit answers [40]. For complex search spaces (with multiple local maxima) there is not a direct correlation between population size and the fitness of the returned answers [41].

Section "Results" reviews the results of the algorithms laid out in this paper, and Section "Discussion" compares these results to a modern SCP algorithm.

## Results

The two databases that the GA and HC algorithm interface with are composed of six separate files which were replicated 100 times and 1000 times. This replication means that there are a predictably finite number of value and weight combinations, which are derived directly from the SDM data. As the sensor and terrain metadata is randomly entered, the Set Covering portion of each mission varies. Each database is loaded completely into memory on the 2 TB Solid State Drive (SSD). The database with 100 missions is 53 GB, and the database with 1000 missions is 346 GB. Table 3 shows the value and weight of the six data logs replicated in the databases:

**Table 3:** Missions value and weight based on SQL queries

Mission	Value	Weight
1	418,653	4940
2	606,444	4743
3	1,036,695	4743
4	694,528	5229
5	712,219	5311
6	231,187	1837

As is discussed in Section "Methods/Experimental", the value is the number of recorded events from the GeodeticPosition (3D) table above 1000 meters and the weight is the total number of s of the mission. While these are specific units, the result of any query could replace these in the algorithm depending on what the researcher is attempting to optimize.

In order to prevent the algorithm from converging prematurely due to a lack of potential higher value answers, the value and weight are randomly altered before being added to the problem data structure. The values of the columns are assigned ± 200,000, and the weights of the columns are assigned ± 1,000 in comparison to what is shown in Table 3. As this adds an additional element of randomness to algorithms which are already stochastic in nature, the Genetic Algorithm and Hill Climber Algorithm are compared on the basis of trends in performance, and not on individual experiments. Each experiment is repeated 10 times.

In general, each algorithm performs as expected. For a given Weight Limit, the Hill Climber Algorithm tends to return less fit answers in a faster time, and the Genetic Algorithm tends to return more fit answers in a slower time. The population size does not produce much variation in returned value. All testing is done on the laptop shown in Table 4:

**Table 4:** Testing equipment and software

Manufacturer	Lenovo
Model	ThinkPad P52 20M9
Processor	Intel(R) Core(TM) i7-8750H CPU @ 2.20GHz
Installed memory	40GB
System type	Windows 10 Pro 64 Bit Processing System
SSD	Samsung SSD 970 EVO Plus 2TB
IDE	Eclipse Version 2019-09 R (4.13.0)
RDBMS	PostgreSQL 11.6

**Fig. 2:**
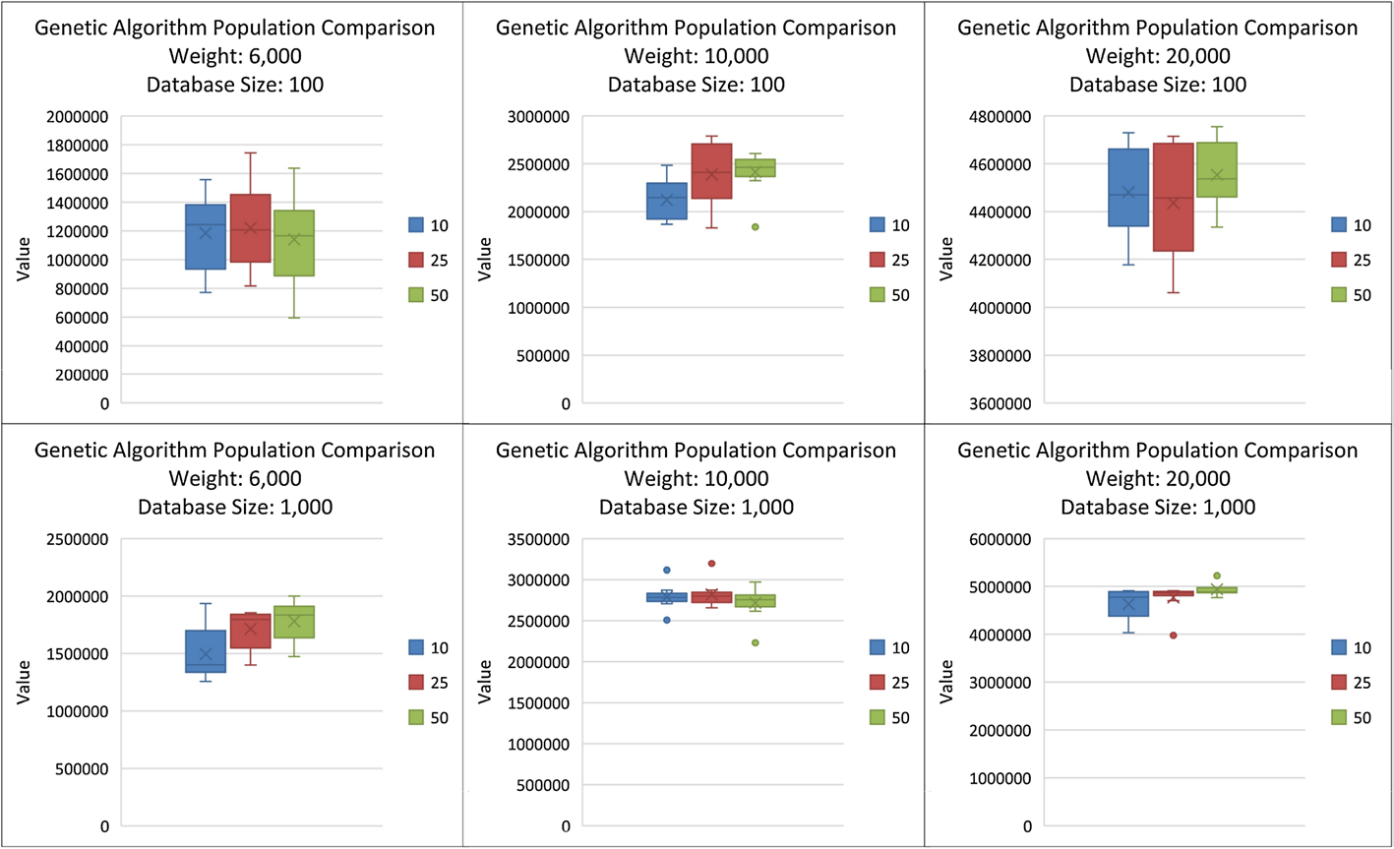
Genetic Algorithm Population Comparison: Compares GA values with populations of 10, 25, and 50 with Databases of sizes 100 and 1000

Both the Genetic and the Hill Climber Algorithms are tested against Weight Limits of 6,000, 10,000, and 20,000, and against the databases with 100 missions and with 1000 missions. Furthermore, the Genetic Algorithm is tested with populations of 10, 25, and 50 elements. Figure 2 Compares how the GA performed for trials with variations in population and database size.

The population size does not consistently impact the final fitness of the returned solution for a given weight or database size. With a weight limit of 6000 and a database size of 1000, the population of 50 slightly outperforms the population size of 25, while with a database size of 1000 and weight limit of 10,000, the other two population sizes slightly outperform the population size of 50. In general, with the larger database, the variances of the population sizes are more compact and more similar than with the smaller databases.

**Fig. 3:**
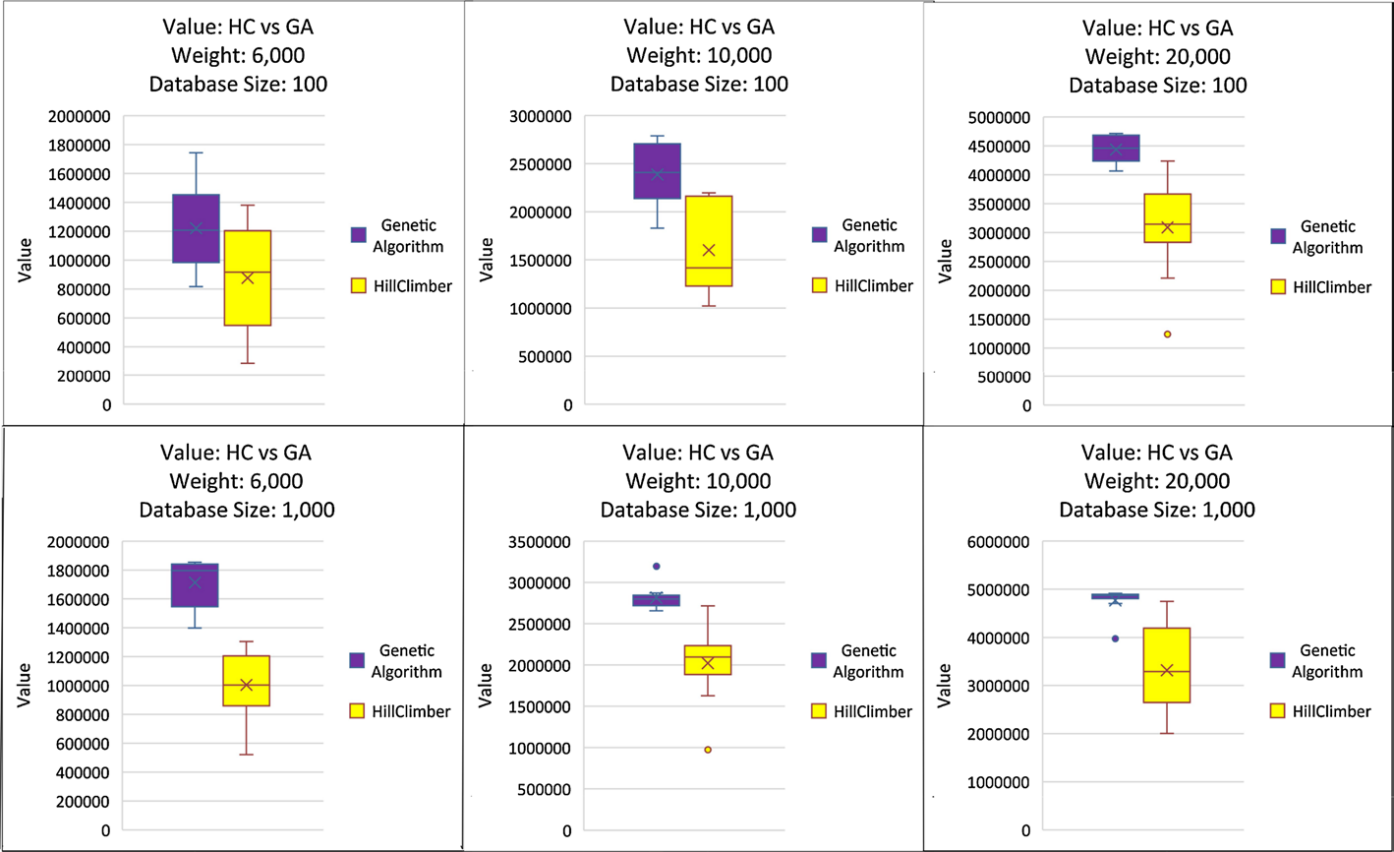
Genetic Algorithm and Hill Climber: Compares GC (Population: 25) Values against HC Values

Figure 3 compares the returned values of the GA against the HC algorithm across ten trials. As expected, the GA overall had a lower variance when compared to the HC and more fit values. The overall fitness of the answers returned by both the HC increased with the larger database, likely because the neighborhoods are larger with more available solutions. The GA also tended to have lower variance and more fit answers with the larger database size. This is likely due to more variety with respect to the higher value files and which sets they are covering.

**Fig. 4:**
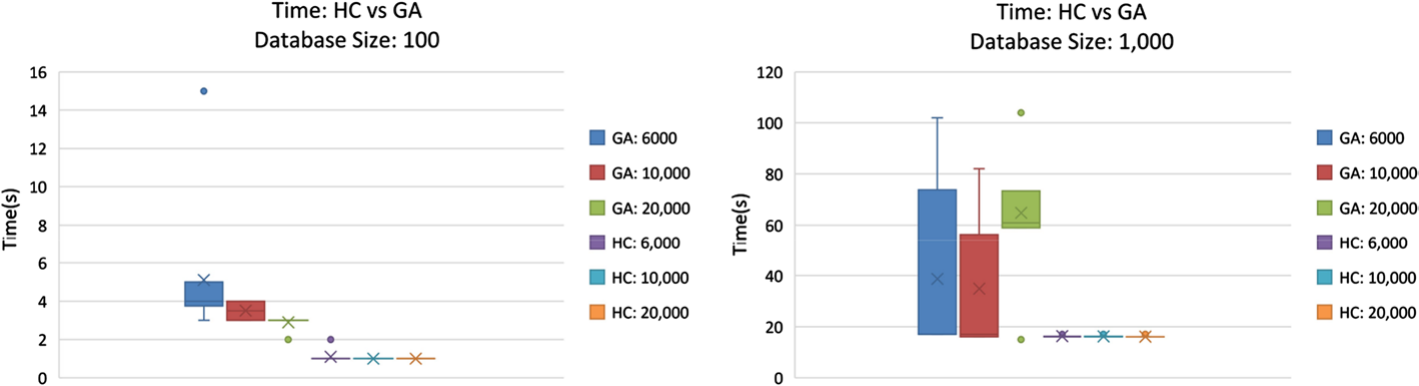
Genetic Algorithm and Hill Climber: Compares GC (Population: 25) Times against HC Times

Figure 4 shows the time and variance of the GA and HC algorithm. The HC finished in under 2 s for the 100 mission database and around 17 s for the 1000 mission database. Most of this is due to the overhead completing the SQL queries listed in Section "Methods/Experimental", and not due to performing the HC algorithm. The GA ranged between 18 s and just over 100 s.

Table 5 shows the average number of solutions found for the HC and GA. As expected, the GA found more solutions than the HC, and both algorithms found more solutions when going from the database with 100 missions to the database with 1000 missions.

**Table 5:** Number of Solutions Found for HC and GA algorithms (Population: 10)

Algorithm	GA	HC	GA	HC	GA	HC
Weight Limit	6000	6000	10,000	10,000	20,000	20,000
Average Number of Solutions Found: 100 Mission Database	793	2	11034	2	72751	4
Average Number of Solutions Found: 1000 Missions Database	4411	4	91003	4	388705	7

## Discussion

As the Combined KP/SCP problem domain is original, there are not any comparable algorithms available in literature which solve this exact problem. Furthermore, this problem is unique in that it is built around a series of database queries, which are based on a specific database schema populated with both repeated and randomized data. However, it can be compared to algorithms which solve similar problem domains. One such algorithm is the BCSO algorithm, which is designed to work with the SCP PD, and is detailed in [9]. They use sets of standardized SCP data to test their algorithm, reporting the average time and value [42]. In order to show the quality of their solutions, they use Percentage Deviation Relative (RPD) to compare the average value of their answer against the known optimal solution for the SCP.

$$\begin{aligned} RPD = \frac{Value_{average} - Value_{optimal}}{Value_{optimal}}*100 \end{aligned}$$

This same equation is used with the results of the GA and HC to get as much of a fair comparison as possible between the approaches. Due to the randomized nature of the data presented in this paper, there is no known optimal value for the combined KP/SCP. Table 3 provides the values and weights of the various columns, with the caveat that values are changed by ±200,000 and weights are changed by ±1000. Assuming a weight limit of 20,000 and four instances of Mission 3 being available in the optimum solution with each mission having a bonus of 200,000, the optimum answer would be 4,946,780. This is assumed to be the optimal value for comparison. Note that the true optimum value could potentially be higher or lower than this value for a given test case. Experimentation showed that both the HC and GA algorithms took about 16 s to complete the queries listed in Section "Methods/Experimental" and to populate their data structures. For simplicity, it is assumed that the BCSO would also take 16 s to complete these queries, and so 16 s was subtracted from the average times for the GA and HC results shown in Table 6.

**Table 6:** BCSO Comparison to GA and HC

	Average Time (s)	RPD
GA	49.8	3.59
HC	0.8	32.9
BCSO	4.55	9.73

Table 6 demonstrates the difference in performance between the BCSO and GA and HC algorithms. The BCSO is faster than the GA, with inferior returned values, and slower than the HC, with superior returned values. This is perhaps an apples to oranges comparison, as they operate within different problem domains, operating on different data, and it would be difficult to know exactly how well the BCSO would would perform if it were converted to the combined KP/SCP PD and made to solves queries for the listed databases. This is an indication, however, that the GA and HC algorithms are at least competitive with a modern algorithm, depending on preferences for speed vs quality of the returned solution.

## Conclusion

This paper demonstrates two algorithms which successfully return solutions to the additive MO KP/SCP problem query. The Genetic Algorithm GA uses a binary encoding, a k-ary tournament selection, crossover, mutation, and feasibility functions to continuously develop new solutions from a population. New solutions are tested for fitness with respect to the population, and are added back in replacing a less fit member. The algorithm concludes if a certain amount of time has passed or if the most fit member of the population has not changed after 60 iterations of the genetic algorithm. The Hill Climber HC algorithm uses the binary encoding from the GA, and generates a single valid solution. The algorithm then flips every 0 and 1 in its solution (searching the neighborhood), and checks for more fit solutions. The algorithm concludes when all 0s and 1s are flipped.

For the GA, differences in population size did not consistently make a difference in performance. This may be due to the relatively small size of the databases and the repetitive nature of the data. When comparing the GA and HC algorithms, the GA consistently gives answers of higher value, and the HC algorithm consistently returns answers more quickly. This difference is more pronounced with the larger database. The results for both algorithms were compared to the modern BCSO algorithm. Correcting for the time to perform queries and populate data structures, the HC returned less optimal results in a faster time and the GA returned more optimal results in a slower time. This is not a perfect comparison, as the combined KP/SCP PD is different from the SCP PD, but it is an indication that the results demonstrated in this paper are competitive with a modern SCP algorithm.

During testing, the SQL queries are repeated for each rendition of the algorithm. In practice, this is not necessary, and for especially time-consuming SQL queries, the queries could be run one time and the algorithms multiple times returning each answer based on the users preference. The algorithms used here could be applied to any RDBMS, as long as the queries return data which match the format described in the algorithms (i.e. a set number of type categories and values distinguished by the user and translated into SQL). Thus the power of these algorithms is that relatively simple queries return more complex results in the KP/SCP PD.

These algorithms and their associated SQL queries are implemented on two databases which exist entirely on the test laptop’s SSD. The final implementation of the AFIT PNT database will be distributed across multiple nodes, due to the anticipated growth of the database. While this may impact query speed, it will not impact algorithm performance, as the algorithms will be implemented as part of a programming language such as Java or C++ which will be accessible to researchers through a User Interface.

There is significant future work for each algorithm implementation. As the database is implemented and goes live with SDM data and its associated metadata, these algorithms should be tested and compared with these operational data sets. Furthermore, the code should be rewritten to make it more useful friendly. Allowing researchers to write their own queries which could populate the problem data structures, and to choose an arbitrary number of row types, could potentially enhance the database usefulness, and may lead to the possibility of the algorithms being used with other databases. Another possibility would be to add additional value categories, and to develop a way to prioritize and optimize between them.

Furthermore, both the HC and the GA should be kept available, as some filter researchers may prefer solutions which are more fit and are willing to wait for them, and others may desire solutions which are faster and are willing to accept less fitness. The desired solution fitness may vary based on the specific context of a problem, and multiple runs of the GA and HC may be necessary to find data sets that meet specifications. While both algorithms appear to scale well for databases composed of repeated files and randomized metadata, it is impossible to know precisely how they will perform with varied files and non-randomized metadata without testing them in this context. How the HC and GA perform for these more interesting and realistic problems should be researched as they become available, and the algorithms updated as necessary.

Finally, researchers should continue to look for applications of the classical SCP and KP PDs and their respective algorithmic solutions in Big Data. This paper laid out two stochastic algorithms and a specific RDBMS, but there are many additional PDs and associated algorithms that could be of value, and Big Data databases where they could be used. In general, the concept of using simple queries to place data into data structures in order to create sophisticated results would have many applications. Ultimately the use cases should come from researchers who are using the databases, in conjunction with proficient computer scientists who can fit the use cases with the correct equivalent PDs and algorithms, so that research is improved.

## Data Availability

All data used in this paper is available on data.mendeley.com under 10.17632/ppd7np4dm6.1. Sean Mochocki is the contributor.
